# People Reject Free Money and Cheap Deals Because They Infer Phantom Costs

**DOI:** 10.1177/01461672241235687

**Published:** 2024-04-08

**Authors:** Andrew J. Vonasch, Reyhane Mofradidoost, Kurt Gray

**Affiliations:** 1 University of Canterbury, Christchurch, New Zealand; 2 University of Milano–Bicocca, Italy; 3 The University of North Carolina at Chapel Hill, USA

**Keywords:** phantom costs, incentives, hidden motives, incentive backfires, irrationality

## Abstract

If money is good, then shouldn’t more money always be better? Perhaps not. Traditional economic theories suggest that money is an ever-increasing incentivizer. If someone will accept a job for US$20/hr, they should be more likely to accept the same job for US$30/hr and especially for US$250/hr. However, 10 preregistered, high-powered studies (*N* = 4,205, in the United States and Iran) reveal how increasing incentives can backfire. Overly generous offers lead people to infer “phantom costs” that make them less likely to accept high job wages, cheap plane fares, and free money. We present a theory for understanding when and why people imagine these hidden drawbacks and show how phantom costs drive judgments, impact behavior, and intersect with individual differences. Phantom costs change how we should think about “economic rationality.” Economic exchanges are not merely about money, but instead are social interactions between people trying to perceive (and deceive) each others’ minds.

## Significance Statement

This article introduces the concept of “phantom costs,” which explain why incentives backfire. This effect is important for any situation in which incentives are offered, ranging from jobs to governmental policies. The standard model in economics assumes people respond rationally to incentives—for example, people are more likely to accept offers for more money than less money. However, this reveals that people spontaneously appreciate the social context of financial offers—especially overly generous offers. Phantoms costs reveal an important change from the standard model beyond standard heuristics and biases. Phantom costs also provide a framework to make sense of other seemingly paradoxical effects of money and provide an important bridge between behavioral economics and social cognition.

## People Reject Free Money and Cheap Deals Because They Infer Phantom Costs

The “price effect” is a central assumption of rationality in economics: Buyers should like low prices and sellers should like high prices (Frey, 1997; Simon, 1955). But here we reveal a systematic reversal of the price effect, documenting a family of situations where offering more money backfires—making people *less* willing to agree to economic transactions. This backfiring occurs because of “phantom costs”: When someone receives an overly generous offer, they imagine hidden downsides to explain this surprising generosity. The backfiring of more money seems to suggest that people are irrational, echoing claims of many behavioral economists. But we argue that people are actually being quite rational, if we broaden our understanding of economic exchanges as social interactions—extending the insights from psychology into economics.

Economics long understood people as rational agents who efficiently seek to maximize gains and minimize losses (Frey, 1997; Simon, 1955). Insights from the cognitive psychologists Tversky and Kahneman convincingly revealed that people are not fully rational (Kahneman et al., 1991), oversimplifying decisions with heuristics (Tversky & Kahneman, 1974), and weighting potential losses more than potential gains (Kahneman & Tversky, 1979). Countering the claims of irrationality, other cognitive psychologists argue that relying on heuristics makes sense because they are efficient (Gigerenzer & Goldstein, 1996; Rangel et al., 2008)— making the best of humans’ limited cognitive resources.

This back and forth between psychologists suggests that questions of rationality depend upon the context. What may be irrational within a narrow economic context can seem more rational in a broader sense (Grossmann & Eibach, 2021). Here, we suggest that people may act “economically irrationally” by not valuing extra money—accepting moderately generous offers but denying extremely generous offers—but this behavior is reasonable if we consider the broader social context of economic transactions. Economic transactions are often modeled using abstract concepts like supply and demand—but there are agents behind every transaction—agents with intentions, motivations, and desires (Child, 2020). When seeking to buy something, people do not merely engage in impersonal “economic cognition,” but also engage in *social cognition*, appreciating the broader interpersonal considerations surrounding pricing. People implicitly understand economic transactions as a duel between strategic and self-interested parties, who may be trying to exploit or deceive the other side.

### Money and Minds

People are intuitive psychologists who read thoughts and motivations into behaviors (Fiske & Taylor, 1991; Frith & Frith, 2005; Malle, 2011; Premack & Woodruff, 1978; Ross, 1977; Tetlock, 2002; Tomasello et al., 2005; Wegner & Gray, 2017; Zadny & Gerard, 1974). Whether in romantic relationships, friend groups, or economic transactions, people understand that others usually have reasons for their behaviors. Economic transactions are special in that they are seen as economically self-interested, where everyone is trying to maximize their benefits and minimize their costs. Of course, there are many motivations within transactions, including preserving face (Falck & Heblich, 2007; Le Grand et al., 2021), seeming magnanimous (Trigg, 2001; Wilcox et al., 2009), and acting ethically (Auger et al., 2008), but everyday people endorse the “norm of self-interest,” expecting others to try their utmost to get the most money for themselves (Miller, 1999).

When someone is more generous than seems warranted, people should wonder “why?” and seek out an explanation (Ratner & Miller, 2001). The norm of self-interest rules out the explanation that people are just feeling generous and suggests another darker explanation—that people are trying to hide some “phantom costs” (Ratner & Miller, 2001). People imagine phantom costs because they would explain why the overly generous offer was made—the offeror knows it’s not actually overly generous. Most obviously, they could be hiding that a product is low quality (Akerlof, 1970; Mastrobuoni et al., 2014; Verma & Gupta, 2004), but phantom costs could be any kind of hidden risk in the transaction known to the offeror that would explain why they are trying to sweeten a bad deal.

Some research finds that participants infer hidden risks when researchers offer overly generous payments in their studies (Cryder et al., 2010). Of course, most people know that researchers are checked by the IRB, but in general situations of economic exchange, people may be afraid that overly generous offers are a trap. The offeror may wish to harm the other person, use them to further an ulterior agenda, or curry future favors from the offeree (e.g., Ouyang et al., 2018) knowing that most people feel indebted by norms of fairness to reciprocate generosity (Bicchieri, 2008; Frey & Bohnet, 1995; Hoffman & Spitzer, 1985). Altogether, when people are given an overly generous offer, they will assume it’s “too good to be true” and believe they are being exploited.

There are some situations where people are less likely to infer phantom costs: When people infer a more benevolent reason for atypical generosity. This reason must be sufficient to explain the generosity: For example, someone is generous to their mom, the offeror is ignorant of the value of what they are offering, or the offeror is dispositionally unselfish (e.g., they took a religious vow of poverty). But in typical transactions, these would not be the case. Moreover, some people will be more likely to infer phantom costs when the situation is ambiguous. Highly suspicious people will imagine more; credulous people will imagine less. Greedy—or desperate—people may also neglect phantom costs because they fixate on the money, like why scammers prey on the desperate.

Altogether, we propose three criteria to produce phantom costs. First, a norm of self-interest applies—as is typical of economic transactions. Second, one party violates that norm of self-interest. Third, no other salient reason explains why they violated the norm. When all three criteria for the context are satisfied, most people should robustly imagine phantom costs, and when the criteria are not fully satisfied, people will imagine them to a lesser degree, if at all. Importantly, norms and context influence when an offer seems normal versus “overly generous,” therefore likely to prompt the perception of phantom costs. Offering US$100/hr for a job when US$10/hr is typical would be more suspicious than offering US$30/hr—but US$30/hr is still somewhat suspicious. Likewise, where the norm is to pay US$0, offering any amount of money should generate phantom costs. The deviation above the norm produces phantom costs—not the absolute amount of money offered.

### Phantom Costs Can Make More Money Backfire

The inference of phantom costs explains when offering more money backfires—when offering more makes people less likely to do a transaction. Standard economic models assume monotonicity: If people will accept an offer for *x* dollars, they should be more likely to accept an offer for *x* + *n* dollars, where *n* > 0 (Frey, 1997; Simon, 1955). Monotonicity means increasing the value of an offer should increase acceptance of it, or at least increasing value should never reduce acceptance. Of course, all things being equal, people do prefer more rather than less money (Gibbons, 1998; Lazear, 2000; Lee & Puranam, 2017; Prendergast, 1999), but we suggest that excessive amounts of money create an economic context—at least in people’s imaginations—where all things are *not* equal—they imagine phantom costs.

When costs rise—even when costs are imagined—transactions become less appealing. Thus, even though extra money increases the *economic* value of the transaction, it can create a net reduction in the transaction’s *psychological* value if it produces too many phantom costs. If someone wants to pay you an extra dollar, but you imagine two dollars of phantom costs come with it, the extra money in the offer reduces its net psychological value. Thus, what appears to be an irrational decision—to reject an offer because they offered more money—is perfectly rational in the context of social cognition.

The money backfiring effect of phantom costs is somewhat like other “backfire effects” in the behavioral change literature, where a nudge or policy manipulation can have the opposite of its intended effect; However, it differs in that those effects occur via different mechanisms including “reputation concerns, shame, feelings of increased accountability, or a change in social norms” (Bolton et al., 2020). For example, charging parents a fine for being late to pick up their kids from daycare can make them *more* likely to be late—because when they pay for it, it no longer feels like a moral failing to be late (Gneezy & Rustichini, 2000). Phantom costs, however, cause more money to backfire through changing the perceived costs and benefits of a potential transaction, not through moralizing it.

Money backfires for phantom costs are also different from the “overjustification effect,” where offering people money or other extrinsic rewards to do enjoyable tasks decreases their motivation to do them (e.g., paying kids for every A+ makes them less intrinsically motivated to do well in class; Deci, 1971; Lepper et al., 1973; Tang & Hall, 1995; Weibel et al., 2014). Economists have applied the overjustification hypothesis to explain why money sometimes “crowds out” intrinsic motivations to donate time, money, or blood (Frey & Oberholzer-Gee, 1997; Müller & Rau, 2020; Titmuss, 1970). Deci and Ryan (2012) argue that people can feel as though their autonomy is undermined by extrinsic rewards that influence or coerce them into doing things, and this threat to autonomy reduces the intrinsic motivation to do those things. By contrast, Lepper and Greene (2015) argue overjustification influences self-perception: People think they must have solved the puzzles to get the reward, and thus they learn about themselves that they were not intrinsically motivated to solve them.

There are three key differences between the overjustification effect and phantom costs. First, overjustification stems from the reward of money, rather than the offer; whereas with phantom costs it is the *offer* of money—even if the offer is rejected—that reduces interest in the transaction. Second, overjustification saps intrinsic motivation when contingent rewards are offered, whereas phantom costs do not sap intrinsic motivation to do the thing—someone paid to eat ice cream will still be motivated and tempted to eat it, but they may refuse the ice cream because they assume it’s tainted. By contrast, a person experiencing overjustification could refuse the ice cream because the money reduces its intrinsic appeal (an effect that would surprise us if it were true). Third, overjustification is meant to explain a loss of motivation for intrinsically motivated activities (Lepper & Greene, 2015; Weibel et al., 2014). By contrast, phantom costs predict backfire effects even when people are not intrinsically motivated.

More broadly, phantom costs relate to social cognitive effects including findings that people are disappointed rather than elated when during price negotiations their initial offer is accepted—because unconditional acceptance implies they could have bargained for more (Galinsky et al., 2002). Similarly, people become surprised when “too good to be true” risk-reward combinations are offered to them, because they expect high rewards to come with risks (Leuker et al., 2019).

### Current Research

Ten experiments tested the hypotheses above in many different situations and sampling from both U.S. and Iranian participants to establish generalizability and using multiple methods to enhance construct validity across different methods. The studies tested monetary backfires stemming from phantom costs when:

1. A stranger offers to pay the participant to do something without sufficiently explaining why they would pay the participant (Experiments 1, 2, 3, 4, 9).
2. An employer offers to pay the participant higher wages than normal for a job without sufficiently explaining why they would pay them extra (Experiments 5-8).
3. A seller offers to sell a product for less money than normal without sufficiently explaining why they would set such a low price (Experiment 10).

This provides a series of tests of the key predictions of our model as applied to economic decisions:

1. People will imagine phantom costs when someone makes an overly generous offer without providing sufficient explanation (Experiment 1-10).
2. Phantom costs will decrease the likelihood of accepting an offered transaction because they reduce its psychological value (Experiment 1-10).
3. Offering money increases the monetary value of a transaction (Experiment 5-7).
4. Offering more money will decrease the likelihood of accepting a transaction whenever doing so reduces the net psychological value of a transaction (Experiment 5-8).
5. Individuals who are especially prone to recognize cues that someone might attempt to exploit them will be more likely to imagine phantom costs when an overly generous offer is made (Experiment 8).
6. People will imagine fewer phantom costs when a sufficient explanation for a generous offer is given, reducing the chances of a monetary backfire effect (Experiment 9-10).

We report how we determined our sample size, all data exclusions (if any), all manipulations, and all measures in the studies. All experiments were approved by human ethics at UNC Chapel Hill or University of Canterbury or Islamic Azad University. Hypotheses and data analysis were preregistered. All data, materials, and preregistered hypotheses are available: https://osf.io/jp7td/?view_only=404b25832d2e49819321d00dfd9fc286.

## Money Backfires From Offering Money When None Should Be Offered

Experiments 1 to 4 tested phantom costs when a stranger offers a monetary incentive to get the participant to do something they might do for free. In three of the situations, the stranger offered participants a favor *and* to pay them to accept it: offering payment for the other person to eat a cookie, to eat ice cream, or to accept a ride home with them from the airport. In a fourth situation, the stranger offered to pay them for a minor favor: opening a door for them. We predicted offering money would generate phantom costs, reducing the net psychological value of the offer, and therefore backfire by *reducing* the likelihood they would accept the offer. Results supported this hypothesis in each study, see Table 1. Each experiment measured phantom costs in a different way to minimize experimenter demand and establish measurement validity. Table 2 contains the measures for each experiment.

**Table 1.** Design and Results of Experiments 1 to 4.

Study	Context	Type of generosity	Condition	Phantom costs			Money backfire	Behavior		
1	Offering cookies to strangers	Paying US$2 vs. no payment	Overly generous	Not measured			Payment reduced eating cookies	20.5% ate the cookie	χ^2^ = 8.33 (*df* = 1, *N* = 182) *p* = .004	*V* = .21
			Not overly generous					39.4% ate the cookie		
2	Offering ice cream to strangers	Paying US$100 vs. no payment	Overly generous	*M* = .71, *SD* = .46, 95% CI = [.62, .80]	*t*(197) = 3.66, *p* < .001	*d* = .52	Payment reduced eating ice cream	*M* = −.79, *SD* = 2.53, 95% CI = [−1.24, −.34]	*t*(199) = 3.42, *p* < .001	*d* = .49
			Not overly generous	*M* = .46, *SD* = .50, 95%CI = [.36, .55]				*M* = .35, *SD* = 2.05, 95% CI = [−.11, .82]		
3	Opening a lobby door for a stranger	Paying US$100 vs. no payment	Overly generous	*M* = 1.79, *SD* = 1.11, 95% CI = [1.54, 2.04]	*t*(199) = 12.74, *p* < .001	*d* = 1.81	Payment reduced opening the door	*M* = −2.16, *SD* = 1.38, 95% CI = [−2.48, −1.84]	*t*(199) = 7.58, *p* < .001	*d* = 1.07
			Not overly generous	*M* = −.48, *SD* = 1.40, 95% CI = [−.73, −.23]				*M* = −0.41, *SD* = 1.38, 95% CI = [−.73, −.09]		
4	Accepting a ride home from a stranger	Paying US$100 vs. no payment	Overly generous	*M* = 1.87, *SD* = 1.51, 95% CI = [1.56, 2.19]	*t*(197) = 4.77, *p* < .001	*d* = 0.68	Payment reduced accepting the ride	*M* = −1.69, *SD* = 1.81, 95% CI = [−2.06, −1.32]	*t*(197) = 4.19, *p* < .001	*d* = .60
			Not overly generous	*M* = .79, *SD* = 1.67, 95% CI = [.48, 1.11]				*M* = −.58, *SD* = 1.92, 95%CI = [−.95, −.21]		

**Table 2.** Vignettes, Measures, and Most Common Phantom Costs for All Experiments.

Experiment	Context	Money	Phantom costs	Extrinsic motivation	Money backfire	Common phantom costs in open-ended responses
**1: Paid to eat cookies**	“Hi, excuse me, I’ve been just been having food with my friends and we had lots left over so I am just trying to get rid of these cookies, would you like one?”	He offers to pay US$2 if they eat a cookie	Not measured	Not measured	The percentage of participants who took a cookie and ate it.	Not measured
		He does not offer money				
**2: Paid to eat ice cream**	You are in the park relaxing on a hot summer’s day. A group of five guys in their 20s are just packing away their picnic cooler when one of them comes over and asks if you would like a spare ice cream sandwich. “We don’t want it to melt and go to waste,” they say.	They offer US$100 if they eat the ice cream	Open-ended “Please briefly explain your answer.”	Not measured	“I would eat the ice cream sandwich” (−3 Strongly disagree to +3 Strongly agree)	Something wrong with the ice cream (e.g., dirty, poison, drugged)
		They do not offer money				
**3: Paid to let someone in a lobby**	You are just walking up to your apartment block when you come across a person waiting outside looking like they need to get in. They ask if you would mind letting them in. “I am just visiting a friend,” they say, “they said they would come down to let me in.”	He offers to pay US$100 to let him in	“Why do you think the person wants to come in?” (Open-ended response) The person has good intentions for coming in.” “The person is visiting a friend.” “The person is a good person.” (−3 Strongly disagree to +3 Strongly agree)	Not measured	“You would open the door for them” (−3 Strongly disagree to +3 Strongly agree) Likert scales.	Robbery, hurt/kill someone, unspecified sketchiness
		He does not offer payment				
**4: Paid to take a ride home**	You are almost home after a long flight. The flight was delayed so you have missed the last bus from the airport; however somebody else on your flight offers you a ride home. “My friend is coming to pick me up and we have a spare seat,” he says.	He offers to pay US$100 to let him drive you home	Participants wrote an open-ended response to the prompt “Please briefly explain your answer.” On the next screen, participants answered “I would be scared to take the ride” “It would be dangerous to take the ride” and “I would worry about taking the ride” (−3 Strongly disagree to +3 Strongly agree)	Not measured	“I would take the ride” (−3 Strongly disagree to +3 Strongly agree)	Danger, human trafficking, kidnapping
		He does not offer payment				
**5: High wages to drive trucks**	You are a freelance truck driver who makes an average of US$20/hour. While looking for work on craigslist, you come across an advertisement seeking someone to drive a truck from one part of the country to another. It reads, “We are seeking a truck driver. We will pay you US$X/hour for the job	The normal wage for a truck driver is US$20/hr. This job paid a randomly assigned amount from US$20-US$250.	There is probably a hidden downside to taking this job. (−10 Strongly disagree to +10 Strongly agree)	Not measured	I would take the job. (−10 Strongly disagree to +10 Strongly agree)	Not measured
**6: High wages to work construction**	You are a freelance construction worker who makes an average of US$15/hr. While looking for work on craigslist, you come across an advertisement seeking someone to help work on a construction site. It reads, “We are seeking construction workers for a job site downtown. We will pay you US$X/hr for the job.”	The normal wage for a construction worker is US$15/hr. This job paid a randomly assigned amount from US$5-US$960.	The job would be extremely hard. The job would be dangerous. There is probably a hidden downside to taking this job. (−10 Strongly disagree to +10 Strongly agree)	I would be really excited by the amount of money. (−10 Strongly disagree to +10 Strongly agree)	“I would take the job.” (−10 Strongly disagree to +10 Strongly agree)	Typo in job ad, scam, job’s in war zone, have to do illegal activity, unspecified risks, stressful/dangerous working conditions
**7: High Wages in a sample from Iran (vignette and questions in Persian)**	You are a freelance cleaner who makes an average of 5,000 t/hr. While looking for work in the newspaper, you come across an advertisement seeking someone to help work cleaning something. It reads, “We are seeking people to clean up a mess. We will pay you X t/hr for the job.”	The normal wage for a construction worker is 5000 *Toman*/hour. This job paid a randomly assigned amount from 2,500 *Toman* to 100,000 *Toman*	There is a hidden downside to this job. The job would be extremely dirty. The job would be disgusting. The job would probably involve illegal activities. This job is dangerous. (1 = Strongly disagree to 7 = Strongly agree)	I would be really excited by the amount of money. (1 Strongly disagree to 7 Strongly agree)	“I would take the job” (1 Strongly disagree to 7 Strongly agree)	Not measured
**8: Individual differences in perceiving phantom costs**	A job advertisement for a construction worker (participants were told to assume they were a construction worker seeing the ad)	The normal wage for a construction worker is US$15/hr. By random assignment, this job paid US$22/hr or US$15/hr.	“The job would be extremely hard,” “The job would be dangerous,” “There is probably a hidden downside to taking this job.” (−10 Strongly disagree to +10 Strongly agree)	I would be really excited by the amount of money. (−10 Strongly disagree to +10 Strongly agree)	“I would take the job.” (−10 Strongly disagree to +10 Strongly agree)	Not measured
**9: Paid to walk a park path**	You are just walking down a path in a public park. A woman coming the other way up the same path says “Keep going, there’s a really nice view down this way.”	She offers to pay you US$20 to walk down that path, but does not explain why	“The woman has good intentions” “There is a nice view down the path” and “The woman is a good person” (−3 Strongly disagree to +3 Strongly agree)	Not measured	Would you go down the woman’s path? (−3 Definitely would not 3 Definitely would)	Something bad, trap, robbery with an accomplice down the path, hide something on the other path
		She offers to pay you US$20 to walk down that path, but explains why (she is on a game show and will win the prize if more people go down that path)				
		She does not offer to pay				
**10: Unusually inexpensive flights**	You are planning a long vacation in Indonesia and are buying a plane ticket from one island to another. The trip should take about 1 hr. You are unfamiliar with any of the local airlines. From the local guidebooks, you expect to pay between US$200 and US$300 for the flight. You find several flights, and three are the best fits for your schedule. Gamma Airlines Flight 3322 US$235 Kappa Airlines Flight 295. US$275 Zeta Airlines Flight 1754 US$X	The third option costs US$15 but it does not explain why it is less	The Zeta flight would be more dangerous than the other flights.” “It would be very risky to take the Zeta flight.” (−10 strongly disagree to +10 strongly agree)	I would be really excited by the cheap price of the Zeta flight (−10 strongly disagree to +10 strongly agree)	I would take the Zeta flight (−10 strongly disagree to +10 strongly agree)	Not measured
		The third option costs US$15 but it explains why: “You read more in your guidebook and learn that the reason Zeta airlines is so cheap is that its seats are very small and uncomfortable”				
		The third option costs US$205				

### Experiment 1: Paid to Eat Cookies

A man approached people on a university campus offering either a cookie alone or a cookie plus two dollars. We predicted a money backfire: Offering money to sweeten the deal would instead reduce the chances they would eat the cookie because they would imagine phantom costs.

#### Method

A student research assistant, “Tom,” dressed plainly, and always carrying a clear plastic container containing about half a container’s worth of chocolate-chip cookies, approached 200 strangers on a university campus and said “Hi, excuse me, I’ve just been having food with my friends, and we had some cookies left over. [Would you like one?/I’ll pay you US$2 to eat a cookie.]” (We initially preregistered a 100-person sample but recruited an additional 100 people to increase power). To avoid biased selection of participants and tipping people off, Tom went to a different area of campus for each trial and approached the first eligible person he saw: adults sitting alone outside. If a participant took a cookie, Tom would leave, but surreptitiously observe whether they ate the cookie. Afterward, Tom asked participants about their decision and debriefed participants. Eighteen participants were excluded for misunderstanding the offer (5), dietary restrictions that prevented them from eating cookies (9), or believing that the offer was part of a psychology study (4).

#### Results

As predicted, participants were significantly less likely to eat the cookie in the payment condition (20.5%) than in the no payment condition (39.4%), χ^2^(1, *N* = 182) = 6.32, *p* = .012, *V* = .21, see Table 1. Only three people took the cookie but did not eat it. Participants were also less likely to take a cookie in the payment condition (22.9%) than the no payment condition (41.4%), χ^2^(1, *N* = 182) = 7.58, *p* = .006, *V* = .20. Thus, offering money backfired: People ate fewer cookies when offered payment. Before debriefing, many participants explained their decision: They frequently stated they thought something was wrong with the cookies, especially if they had decided not to eat a cookie.

### Experiment 2: Paid to Eat Ice Cream

The next three experiments built on the findings of the cookie study to establish the mechanism behind why people rejected the cookies. Experiment 2 offered online Mechanical Turk participants a vignette about a similar situation and asked not only what they would do, but why. In Experiment 2’s vignette, a stranger at a park offered them an ice cream sandwich and by random assignment either offered to pay them US$100 to eat it or did not offer money.

#### Method

Two hundred participants rated “I would eat the ice cream sandwich” on a (−3 Strongly disagree to +3 Strongly agree) Likert-type scale. Participants responded to an open-ended prompt merely asking them to explain their decisions—thereby measuring potential phantom costs while minimizing any potential for experimenter demand to drive this effect.

Two independent coders blind to condition-coded responses for mention of risk. Disagreements in coding were resolved by discussion until coders were in complete agreement. Open-ended responses including phantom costs were coded “1” and others were coded “0.” For example, one participant (coded 1) wrote “Everything was fine about getting a free ice cream sandwich until the $100 was offered. That is too much money for something not to be fishy about the offer.”

#### Results and Discussion

Payment reduced the likelihood of eating it, see Table 1; that is, money backfired.

Participants were generally wary of strangers giving away free ice cream, but they were more likely to report risk in the US$100 condition than in the free condition, see Table 1. This result is consistent with phantom costs explaining this money backfire, but inconsistent with an overjustification explanation. If it were overjustification, people should have reported disinterest in the food, rather than worries about costs such as it being tainted or contaminated.

Mediation analysis (Iacobucci, 2012) found phantom costs mediated the difference in rates of eating the ice cream between conditions, *Z*_mediation_ = −3.50, *p* < .001. Thus, offering money to someone to eat ice cream made them less likely to eat it because people imagined phantom costs.

### Experiment 3: Paid to Let Someone Into a Lobby

Experiment 3 (*n* = 201 Mturkers) extended this research by using Likert-type scales to measure phantom costs to help establish this measurement approach. We also used a new vignette about a different situation to enhance generalizability: A stranger asked participants to let them into a lobby, and by random assignment either offered US$100 or no money to let them in. To minimize experimenter demand, we purposefully measured the opposite of phantom costs, using disagreement with positive items as our measure of phantom costs: For example, disagreement with “The person has good intentions for coming in.” We also measured level of agreement with “You would open the door for them” (−3 Strongly disagree to +3 Strongly agree).

As predicted, offering payment made people less likely to let them in, *t*(199) = 7.58, *p* < .001, *d* = 1.07, see Table 1. People imagined more phantom costs when they were offered payment, *t*(199) = 12.74, *p* < .001, *d* = 1.81, and higher phantom costs mediated decisions to not let them in *b* = −1.93, *SE* = .25, 95% CI = [ −2.45, −1.48]. Offering money backfired because people imagined phantom costs.

### Experiment 4: Paid to Take a Ride Home

Experiment 4 (*n* = 202 Mturkers) used a new vignette to further enhance generalizability and used both Likert-type and open-ended measures of phantom costs to establish convergent validity. In this vignette, a fellow airplane passenger offered them a ride home from the airport, again by random assignment either offering US$100 to sweeten the deal, or no money. There were three Likert-type measures of phantom costs, for example, “It would be dangerous to take the ride” (α = .95), and open-ended responses—Both measures of phantom costs were highly correlated *r*(197) = .71, *p* < .001, supporting the convergent validity of the measures.

### Results

Money backfired: People were less likely to take the ride when offered money, *t*(197) = 4.19, *p* < .001, *d* = .60, see Table 1. As predicted, offering money increased phantom costs, *t*(197) = 4.77, *p* < .001, *d* = 0.68, and these mediated decisions to not take the ride, *b* = −.92, *SE* = .21, 95% CI = [−1.36, −.52], see Figure 2. Offering money backfired because phantom costs.

### Interim Summary

**Figure 1.**
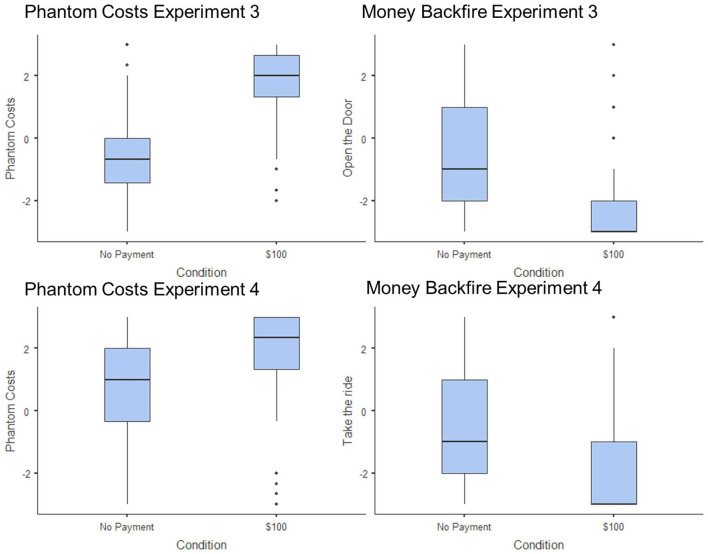
Phantom Costs and Corresponding Money Backfires in Experiments 3 and 4.

Offering money to sweeten a deal backfired across four different situations (Figure 1). In each situation, people were less likely to do what they were asked to do if someone offered to pay them to do it. This happened because people imagined phantom costs.

**Figure 2.**
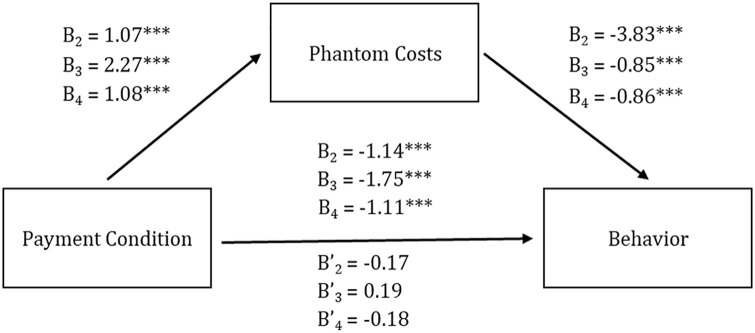
Mediation Pathways, Experiments 2 to 4. Numbers in Subscript Represent Experiment Number.

## Offering Higher Wages Can Backfire

Offering higher wages would normally increase interest in a job—but what if offering higher wages reduces net psychological value because of phantom costs? The next several experiments tested people’s reactions to jobs offering insufficiently explained higher-than-normal wages. Participants read vignettes offering jobs with randomly assigned wages—either below the norm, at the norm, or above the norm for that job.

**Figure 3.**
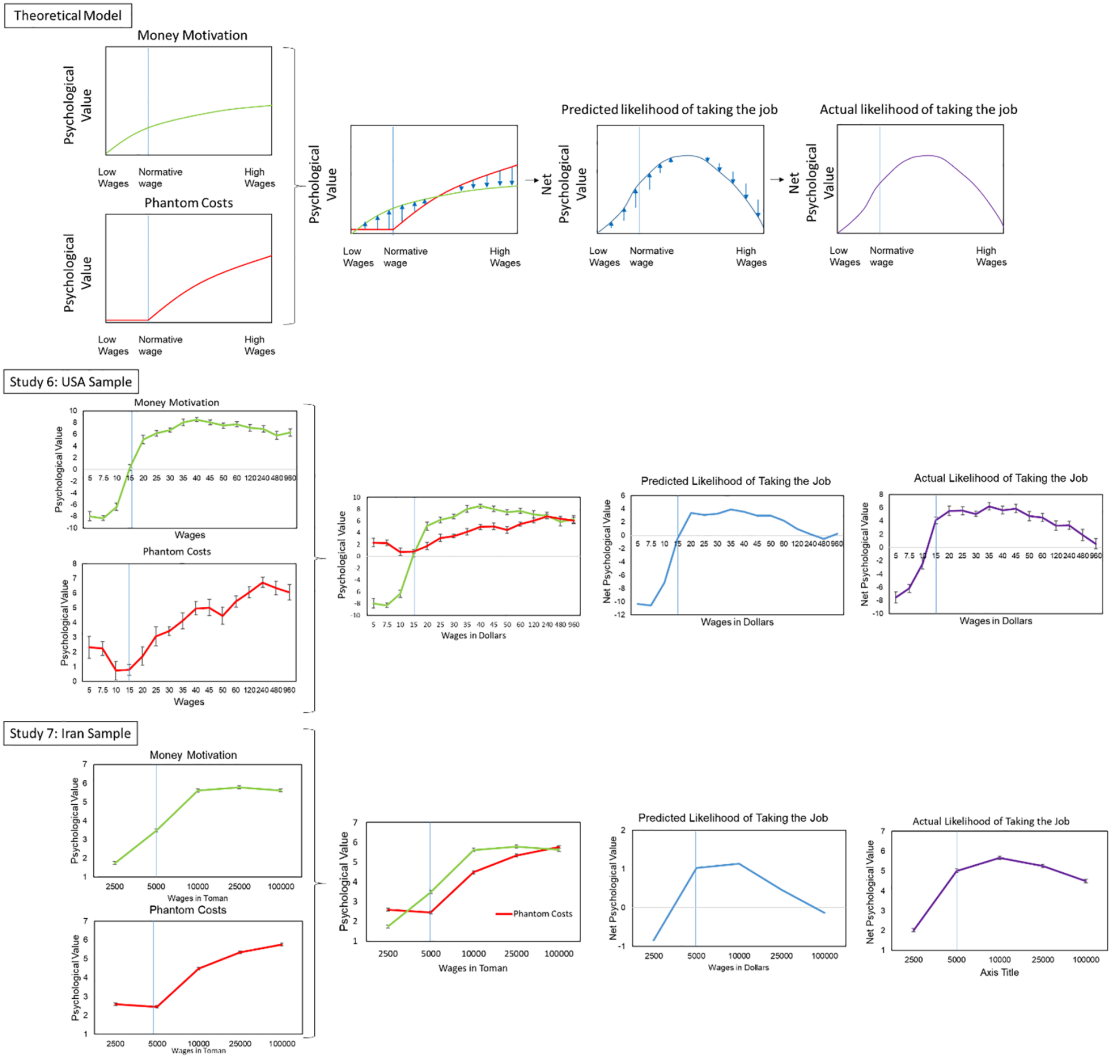
Theorized and Actual Psychological Valuations of Jobs Offering Different Wages. Higher wages increase money motivation and above the normative wages they increase phantom costs. This creates a predictable upside-down *U*-shaped pattern of net psychological value (calculated by extrinsic motivation for the money minus phantom costs). This pattern of net psychological value is our prediction of people’s decisions to take the job at varying wage levels. Normative wages are indicated by a blue line. Error bars indicate standard error.

Increasing wages has two opposing effects, see Figure 3. On the one hand, more money increases extrinsic motivation for the job albeit with diminishing returns because an extra dollar means a lot to someone making very little, but less to someone making a lot (Smith, 1776; *Figure 3, green line*). On the other hand, more money means more phantom costs (*Figure 3, red line*), which are zero below the normative wage and then increase steeply as wages rise above the norm because the more money offered, the worse phantom costs people should imagine. We calculate the net psychological value of an offer by subtracting the phantom costs from the extrinsic motivation. The *blue line* (*Figure 3*) represents the net psychological value of the transaction, which is equal to the *green line* minus the *red line*. This equation produces a characteristic upside-down U pattern. Net psychological value first increases as wages rise, then rises more slowly above the normative wage, before further increases to wages actually decrease net psychological value, because phantom costs rise faster than extrinsic motivation when wages are extremely high. Net psychological value is our predictor of people’s actual decisions about whether to take the job (*Figure 3, purple line*). Our model predicts that money will backfire when wages become so high that net psychological value decreases with each additional dollar offered.

### Experiment 5: High Wages to Drive Trucks

We created a fake job advertisement and informed participants they were a freelance truck driver who typically made US$20/hr. The advertisement offered a randomly assigned wage: either US$20, US$30, US$40, US$100, or US$250. We asked participants whether they would take the job and about phantom costs.

#### Method

861 out of 998 participants (538 women, 321 men, 2 identified as something else) passed an attention check, so were included in final analyses. A sensitivity analysis found 80% power to detect an effect of ρ = .095. Because it was an expensive sample (Qualtrics sample representative of the US on income, region, race, and political party), we asked just two questions: “I would take the job,” and “There is probably a hidden downside to taking this job” on Likert-type scales from −10 strongly disagree to +10 strongly agree.

#### Results

A two-line test (in which an effect is considered curvilinear if it increases in one part of the distribution and decreases in another; Simonsohn, 2018) supported the hypothesis that there would be a curvilinear relationship between wages and job taking. As wages increased from US$20 to US$40, job taking increased (albeit nonsignificantly), *B* = 0.06, *SE* = 0.03, *t* = 1.95, *p* = .051, β = .09. But as wages continued to increase from US$40 to US$250, job taking decreased, *B* = −.02, *SE* = .003, *t* = 5.84, *p* < .001, β = .25. JAMOVI’s General Linear Model (GLM) Mediation model module found a significant indirect effect of wage levels on job taking through phantom costs, *B* = −0.0033, *z* = 4.14, *p* < .001, where higher wages made more phantom costs, which decreased taking the job. Thus, offering a little too much increased job taking, but offering way too much money backfired because of phantom costs.

### Experiment 6: High Wages to Work Construction

Experiment 6 extended this work in several ways: enhancing generalizability by using a different job with a different normal wage, extending the range of wage levels tested, using a more sophisticated index variable of phantom costs, and measuring people’s extrinsic motivation for the money. We predicted that although higher wages will increase phantom costs, they will also increase extrinsic motivation—which should have opposing effects on whether people take the job.

#### Method

Experiment 6 was a preregistered (http://aspredicted.org/blind.php?x=ty8vp8; http://aspredicted.org/blind.php?x=xw5xc2) mega-analysis (Eisenhauer, 2021) of two experiments with 898 total participants testing responses to different wage levels for a job as a construction worker. Sensitivity analysis found 80% power to detect an effect of ρ = .09 or larger. Wage levels varied from US$5/hr to US$960/hr for a job normally paying US$15/hr. Phantom costs were measured via a three-item index (α = .79): “The job would be extremely hard,” “The job would be dangerous,” and “There is probably a hidden downside to taking this job.” Extrinsic motivation for the money was measured: “I would be really excited by the amount of money.”

#### Results

Results supported our model (Figure 3). Money motivation increased with wages, *B* = .0052, *SE* = .00088, *t*(885) = 5.91, *p* < .001, and phantom costs also rose when wages were above the normative level, *B* = .0044, *SE* = .00052, *t*(885) = 8.54, *p* < .001. Crucially, at high wages there was a money backfire effect that closely followed the net psychological value of each wage. A two-line test (Simonsohn, 2018) found that taking the job increased as wages rose from US$5 to US$35, *B* = .42, *SE* = .003, *t* = 16.40, *p* < .001, but decreased as wages then rose from US$35 to US$960, *B* = −.005, *SE* = .0008, *t* = −6.83, *p* < .001. Notably, participants were *less* likely to take a job paying US$960/hr, *M* = 0.58, *SE* = .63, than a job paying US$15/hr, *M* = 4.09, *SE* = .49, *t*(885) = 4.39, *p* < .001.

We tested mediation using JAMOVI’s GLM Mediation Model. As predicted, higher wages produced two opposing indirect effects: more phantom costs, which decreased taking the job, *B* = −.0014, *SE* = .00026, *z* = 6.08, *p* < .001, and extrinsic motivation for the money, which increased taking the job, *B* = .0037, *SE* = .00064, *z* = 5.75, *p* < .001. Thus, offering high wages backfired because although the financial value increased, so did phantom costs, creating a decrease in net psychological value.

### Experiment 7: High Wages in a Sample From Iran

Experiment 7 extended these findings by testing the phantom cost effect with a different vignette and in a country with a very different culture and different attitudes about capitalism: Iran.

#### Method

505 Iranian participants (287 female, 218 male) were recruited via a social media vehicle in Iran: “Telegram.” Sensitivity analysis found 80% power to detect effects of *f* = 0.05 or larger. We used “cleaner” as the job, because it is a job that both men and women can do in Iranian culture and we expected to recruit both men and women in the sample. The normal hourly wage for a cleaner was 5,000 *toman* (about US$1.19; this is based on a typical wage for this job in Iran in 2020 when the study was conducted), and the job ad offered 2,500, 5,000, 10,000, 25,000, or 100,000. The job ad was translated into Iranian Persian, which was the first language for most participants. Responses to dependent measures were on Likert-type scales from 1 strongly disagree to 7 strongly agree: “I would take the job. I would be really excited by the amount of money. I would feel guilty getting paid this much to clean the mess.” Phantom costs measures (“There is a hidden downside to this job. The job would be extremely dirty. The job would be disgusting. The job would probably involve illegal activities. This job is dangerous.”) were aggregated at each timepoint and were highly reliable (all αs > .90).

#### Results

The pattern of results was similar in Iran and United States (see Figure 3). Repeated measures ANOVAs found phantom costs increased with higher wages, *F*(4,2,016) = 798, *p* < .001, η^2^ = 0.43, but only above the normative wage, and this effect was robust to controlling for age, income, gender, and education. People were more extrinsically motivated by higher wages, *F*(4,2,106) = 648.77, *p* < .001, η^2^ = 0.43. The curvilinear money backfire effect also replicated: People were more likely to take the job as wages increased from 2,500 (*M* = 2.01) to 5,000 (*M* = 5.01) to 10,000 Toman (*M* = 5.67), *t*(504) = 35.94, *p* < .001, *d* = 1.60, but were less likely to take the job as wages continued to increase from 10,000 to 25,000 (*M* = 5.25) to 100,000 Toman (*M* = 4.50), *t*(504) = 10.84, *p* < .001, *d* = .48.

### Discussion

Experiments 5 to 7 supported the predicted curvilinear pattern of job taking due to changes in net psychological value. Below the normative wage, participants were understandably uninterested in the jobs because they were not motivated by the money and they were also concerned the jobs may be of low quality, especially when wages were below minimum wage. By contrast, jobs offering normal wages were neither particularly exciting nor worrying. As predicted, higher wages were both more exciting and worrying. Participants began to imagine phantom costs at slightly higher-than-normal wages. Participants were slightly more likely to take jobs offering up to about double the normal wage. The most intriguing result was participants’ responses to extremely high wages worth several times the normal amount. Participants were extrinsically motivated by the money, but also imagined the jobs came with substantial phantom costs, resulting in a decreased net psychological value compared with the normal wage. Overall, participants were *less* likely to accept jobs offering extremely high wages than normal-paying jobs.

Finding the same pattern in Iran and the United States suggests that phantom costs and a corresponding pattern of money backfires are cross-cultural phenomena not merely found in Western, Educated, Industrialized, Rich and Democratic (WEIRD) countries (Henrich et al., 2010). However, importantly, the backfire occurred at high wages *relative to the normative wage in the relevant social context.* The wages Iranians considered suspiciously high (100,000 Toman/hr was only US$24/hr when the study was conducted) would be less suspicious for the same job in countries with higher normative wages. In addition, because this experiment used a different vignette with different wage levels and different scales, it is possible there might be cross-cultural differences in how people think about phantom costs, but we found mostly similarities.

### Experiment 8: Individual Differences in Perceiving Phantom Costs

The prior experiments manipulated the situation to test whether people would perceive phantom costs from transactions that most people would consider overly generous. This experiment took a different approach. We created a situation that was ambiguous as to whether phantom costs existed and used an individual differences approach. We predicted that people who were characteristically distrusting of others’ potential bad intentions would be more likely to imagine phantom costs in an ambiguous situation. This could mean money would backfire for them more than for others.

#### Method

We tested this using the construction worker wage paradigm from Experiment 6, but with a slightly high wage instead of an extremely high wage, to avoid potential ceiling effects and increase the chance of detecting moderation by individual differences. Distrust was measured using a combination (α = .93) of both the distrust feelings subscale of the General Paranoia Scale for Adults (Barreto Carvalho et al., 2017) and the Interpersonal Exploitation subscale of the Rotter Interpersonal Trust scale (Rotter, 1967).

#### Results

**Figure 4.**
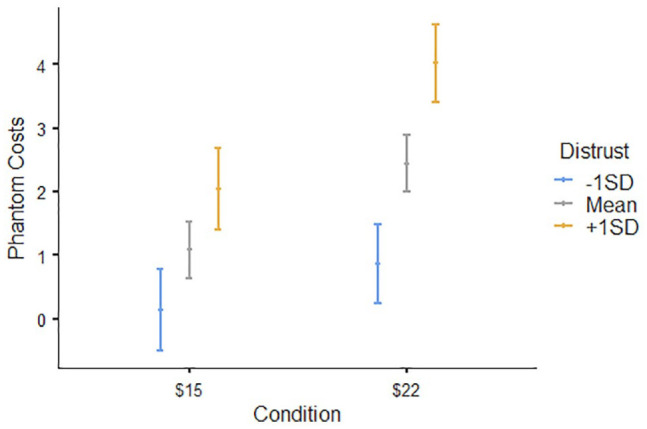
Distrusting Participants Imagined More Phantom Costs Than Trusting Participants, Especially in the High Wage Condition.

The phantom costs and money backfire effects replicated successfully (Supplemental Materials), but crucially, participants high in distrust responded differently than those low in distrust. Distrusting participants were more likely to imagine phantom costs in either condition, *B* = .*76, SE* = *.18, t*(399) = 4.10, *p* < .001, 95% CI = [.40, 1.12], but there was also a significant interaction between distrust and wage level, *B* = .56, *SE* = .25, *t*(399) = 2.23, *p* = .026, 95% CI = [.067, 1.05], which we examined using planned contrasts comparing participants high versus low in distrust. Participants high in distrust (1 *SD* above the mean) imagined significantly more phantom costs as the wages increased, *B* = 2.12, *SE* = .45, *t*(399) = 4.74, *p* <.001, whereas participants low in distrust (1 *SD* below the mean) did not, *B* = .70, *SE* = .45, *t*(399) = 1.57, *p* = .12, Figure 4. Thus, distrusting individuals were indeed more likely to imagine phantom costs, especially when wages were high.

The money backfire effect was especially strong among distrusting individuals. A moderated mediation model (Model 7 of PROCESS; Hayes, 2017) revealed participants high in distrust imagined more phantom costs, especially in the high payment condition, and this mediated their being more likely to refuse the high-paying job, *B* = −.16, *SE* = .09, 95% CI = [−.37, −.01]. Moreover, people high in distrust were also less likely to take a job when they did perceive phantom costs, *B* = −.60, *SE* = .19, 95% CI = [−1.06, −.30], but for participants low in distrust, higher imagined phantom costs did not significantly reduce taking the job, *B* = −.20, *SE* = .15, 95% CI = [−.56,.05]. Thus, being distrusting both increased the likelihood that participants would imagine phantom costs, and made them less likely to take the job when they did imagine them. Moreover, as predicted, distrust’s influence was specific to phantom costs. Distrust did not moderate the relationship between participants’ money motivation and taking the job, *B* = .12, *SE* = .21, 95% CI = [ −.30, 51]. People high in distrust were just as happy as anyone to earn more money—but they were more suspicious that the money came with phantom costs.

#### Discussion

Distrusting people not only imagined more downsides in general but were especially likely to imagine phantom costs when the situation implied them: When wages were unusually high. This means money backfires more often when someone untrusting considers an overly generous offer.

## Reasons for Overgenerosity Attenuate Phantom Costs

Unexplained generosity leads to phantom costs—but what if generosity is explained? Experiments 9 and 10 tested the hypothesis that explaining generous offers would reduce phantom costs and increase people’s willingness to accept a generous transaction. Each experiment had three conditions: standard offer, generous offer without explanation, and newly: generous offer with explanation. We predicted that phantom costs and money backfires would decrease when the offer was explained.

### Experiment 9: Paid to Walk a Park Path

Experiment 9 used a vignette in which a woman sees you in a park and recommends a particular pathway. She either offers to pay you to walk down it or not. In a third condition, she explains why. We predicted that an explanation should reduce the likelihood of imagining phantom costs.

#### Method

Three hundred American Mturkers (182 male, 117 female, 1 undisclosed) read a vignette about a woman recommending a particular park path. In the explained offer condition she explains why: She is a contestant on a game show and will win if you walk down the path. Participants rated “Would you go down the woman’s path?” on a (−3 definitely would not to +3 definitely would) scale. There was a three-item index of phantom costs, all reverse coded to minimize experimenter demand (α = .93): “The woman has good intentions” “There is a nice view down the path” and “The woman is a good person” on (−3 Strongly disagree to +3 Strongly agree) Likert-type scales.

#### Results

Offering money backfired: participants were less likely to walk the recommended path in the unexplained offer condition (*M* = −.56, *SD* = 2.14) than in the no payment condition (*M* = 1.56, *SD* = 1.34), *t*(197) = 8.40, *p* < .001. Crucially, providing a reason for the payment increased people’s willingness to walk the recommended path (*M* = .70, *SD* = 2.08) compared with the generous payment condition, *t*(199) = 3.50, *p* < .001, although they were still less likely to walk than participants offered no payment, *t*(196) = 4.20, *p* < .001.

Phantom costs mediated these decisions. Participants in the unexplained offer condition (*M* = .57, *SD* = 1.63) imagined more phantom costs than participants in the no payment condition (*M* = −1.84, *SD* = 1.18), *t*(197) = 11.95, *p* < .001. Mediation using PROCESS (model 4, with 5000 bootstrap samples; Hayes, 2017) showed phantom costs mediated the money backfire, indirect effect = −2.83, *SE* = .26, 95% CI = [−3.38, −2.33]. Phantom costs also mediated people’s decisions to walk the park more in the justified versus unjustified payment conditions, indirect effect = −1.23, *SE* = .12, 95% CI = [−1.47, −0.99]. Participants imagined less phantom costs in the justified payment condition (*M* = −.13, *SD* = 1.27) than in the unjustified payment condition, *t*(194) = 3.33, *p* = .001, although they did imagine more than in the no payment condition, *t*(197) = 9.87, *p* < .001. Moreover, open-ended responses to the prompt “Why do you think the woman wants you to go that way?” corroborated the pattern of phantom costs using Likert-type scales, for example, “To get pranked. I don’t trust this woman AT ALL.”

### Experiment 10: Unusually Inexpensive Flights

In this experiment, participants read a vignette about different flights in Indonesia (chosen to be unfamiliar to our participants). One of the flights was either a little cheaper or much cheaper than the others. Furthermore, in a third condition, the much cheaper flight was explained. We again predicted the explanation should reduce phantom costs and therefore reduce the backfire effect.

#### Method

301 American Mturkers (10 failed attention check; leaving 169 male, 120 female, 2 other) chose to buy one of three hypothetical flights. In the standard offer condition, the three available flights cost US$235, US$275, and US$205—we expected most people would buy the cheapest option, and they did. In the unjustified offer condition, the options were US$235, US$275, and US$15, and we predicted that fewer people would choose the cheapest flight. In the justified offer condition, the vignette explained that reason the US$15 flight was so cheap is because the seats were very uncomfortable.

#### Results

Money backfired: People were less likely to take the cheapest flight when it cost US$15, *M* = −1.07, *SD* = 7.74, 95% CI = [−2.47,.33], than when it cost US$205, *M* = 5.26, *SD* = 5.43, 95% CI = [3.81,6.71], *F*(1,286) = 38.16, *p* < .001, η^2^ = .118, unless a justifying reason for the US$15 was provided, *M* = 1.44, *SD* = 7.78, 95% CI = [.03,2.86], *F*(1,286) = 6.15, *p* = .014, η^2^ = .021. Thus, providing an explanation moderated the money backfire effect. Notably, uncomfortable seats are usually not a selling-point for a flight, but telling participants about the flight’s bad seats made them *more* likely to take the flight, because knowing about the uncomfortable seats reduced the tendency to imagine even worse phantom costs, such as frequent crashes.

Analyses of phantom costs corroborated this interpretation. As predicted, phantom costs were higher in the US$15 condition (*M* = 3.52, *SD* = 5.53, 95% CI = [2.46, 4.57]) than in the US$205 condition, *M* = −2.49, *SD* = 4.73, 95% CI = [−3.46, −1.52], *F*(1,287) = 20.00, *p* < .001, η_p_^2^ = .065, and in the uncomfortable seats US$15 condition (*M* = .96, *SD* = 5.66, 95% CI = [−.10, 2.02], *F*(1,287) = 11.26, *p* < .001, η_p_^2^ = .038. Moreover, phantom costs mediated their purchasing decisions, indirect effect = −3.74, *SE* = .73, 95% CI = [−5.32, −2.44].

### Discussion

Providing an explanation for one’s overly generous offer reduces or even eliminates the phantom costs people imagine. In the right situation, this can produce an ironic effect: Revealing a bad aspect of an exchange can make it more appealing because without the reveal people imagine phantom costs that are even worse. For example, revealing an unusually inexpensive flight has uncomfortable seats *increased* people’s interest in buying a ticket because uncomfortable seats are better than the phantom costs people otherwise tend to imagine (e.g., the plane crashes a lot). Experiment 10 also shows that phantom costs arise not just when someone offers to pay you too much money—they also occur when someone asks you to pay too little. Moreover, the results again show large backfire effects due to social cognition: People indicated they would pay *more than 10 times the price* to avoid the cheapest flight because of phantom costs, even though the explicit information about the flights was identical.

## General Discussion

How can you convince someone to buy your product or do work for you? You might attempt to do so by sweetening the deal financially, but these ten high-powered, preregistered experiments suggest that approach could backfire if you don’t explain why the deal is so sweet. People reject offers that seem too good to be true—even if they would have accepted a similar but worse offer. The reason is phantom costs. People imagine phantom costs when people offer them too much without providing sufficient explanation for why. This leads them to reject high paying jobs, inexpensive products, and even free money for doing something they would have gladly done without pay.

The concept of “phantom costs” is pivotal in understanding this behavior. People tend to imagine these phantom costs when they witness a self-interested party being overly generous in an economic transaction without clear reasons provided. Notably, when sufficient explanations were given, people did not imagine phantom costs—at least not to the same extent. The absence of sufficient explanation is a crucial factor in triggering the phantom cost phenomenon. It determines when people are likely to imagine such costs and when offering more money might backfire. Experiments 9 and 10 showed that explaining the reasons for generosity reduced the likelihood of phantom costs, further emphasizing the role of sufficient explanation.

These experiments underscore the necessity of incorporating social cognition into economic decision models. Standard economic theory suggests that lowering prices should increase demand for products and raising wages should attract more workers. However, the concept of phantom costs introduces a new dimension where offering more money can deter people from taking action. This effect can entirely reverse the expected outcome, demonstrating the need for a more nuanced understanding of economic decision-making. Of course, there are several known exceptions to the standard economic model (e.g., Frey & Oberholzer-Gee, 1997; Kahneman et al., 1991), but the phantom costs effect produces a whole new class of situations in which offering more money backfires: whenever phantom costs reduce the net psychological value of the offer.

Phantom costs offer a unique explanation for money backfires. Although previous research has focused on how money can reduce intrinsic motivation (e.g., Deci, 1971), phantom costs operate differently. They stem from the idea that money signals hidden costs within a transaction. Notably, phantom costs can lead to money backfires even when intrinsic motivation is absent—they can cause individuals to reject opportunities they otherwise wouldn’t, simply due to increased rewards.

Future research should explore the underlying mechanisms of other money backfires in the literature. Although motivation crowding has been a commonly proposed mechanism (e.g., Weibel et al., 2014), considering phantom costs as an alternative mechanism is an important future direction. It is possible some prior findings attributed to motivation crowding could be caused by phantom costs. If there are indeed multiple mechanisms at play, this may help explain why money backfire effects are so fickle and situational (e.g., Metcalf et al., 2020).

We propose that phantom costs are part of a broader set of social cognitive phenomena linked by a common mechanism we dub the “heuristic of sufficient explanation”. When insufficient reasons are provided to explain a person’s behavior, people infer that these reasons must nonetheless exist but are hidden. This aligns with other cognitive phenomena, such as people inventing reasons to explain unjustified behaviors. For instance, individuals often assume that a decision-maker who caused a harmful side effect must have a hidden intention to do so (Knobe, 2003)—only when clear justifications for their actions are absent (Rowe et al., 2021; Vonasch & Baumeister, 2017). The same applies to judging political policies involving costly tradeoffs (e.g., cutting taxes versus funding services), where people struggle to imagine sufficient explanations why someone would support policies they oppose, and thus tend to believe the hidden motivation for their opponent’s policies is to cause harm (Goya-Tocchetto et al., 2022). The “phantom cost” effect falls in line with this pattern of attribution, where people infer hidden reasons and motives when clear explanations are lacking (Cusimano et al., 2023; Wong & Weiner, 1981). Future research should explore the broader implications of the heuristic of sufficient explanation.

In several studies, we asked participants to provide an open-ended response articulating their reasons for making whatever decision they made. Table 2 reports the most common phantom costs people mentioned in their open-ended responses (and measures for all studies). The common themes across the various studies were interpersonal threats, for example, the person wanted to rob you, harm you, or kill you. This is interesting because other types of cost were possible (e.g., poor quality product, jobs did not include benefits), but humans are particularly highly attuned to other humans as potential threats (e.g., Maner et al., 2005), and this was supported by exploratory analyses of the open-ended responses. Future research should examine how broadly this pattern generalizes to other phantom costs—are they mainly interpersonal threats?

Would people’s decisions to reject overly generous transactions change if the costs were stated, not phantom? This should depend on the costs involved. Some workers do accept dangerous jobs, and they generally expect higher pay to compensate for the risk (Robinson, 1986). This behavior can be understood using a cost-benefit analysis. So can phantom costs. When someone imagines phantom costs, they count as costs in cost-benefit analysis. Whether those phantom costs are better or worse than the actual cost depends on the situation.

### Limitations and Future Directions

Our model predicts that insufficiently explained overly generous offers may lead to phantom costs, but whether they backfire is a more complex issue, hinging on competing factors. Although people are likely to imagine phantom costs, the extent to which these costs outweigh the perceived benefits of receiving more money varies based on individual judgment and the specific context. Experiments 5 to 7 and the theoretical framework in Figure 3 provide initial insights, suggesting that increasing wages can initially boost job interest but may deter it when extremely excessive compensation is offered.

Another limitation of our research is the use of somewhat unrealistic scenarios in certain vignettes. These exaggerated examples were chosen to ensure the effects were sufficiently pronounced for study purposes, but our findings in Experiments 5 to 8 suggest that phantom costs might manifest even in everyday situations. Some institutions may consciously or unconsciously employ strategies to mitigate the perception of phantom costs; for instance, phantom costs may explain why stores name their sales—a “Black Friday sale” explains price reductions and reduces suspicion compared with unexplained discounts. Moreover, people might imagine phantom costs from non-monetary generosity: for example, when someone offers too much of their time, energy, goods, or help, without sufficient explanation.

We should clarify that the term “phantom costs” does not imply irrational thinking or seeing phantoms; instead, it signifies that these costs are imagined and potentially unreal, though they could also be genuine. People reasonably seek causal explanations for people’s seemingly unjustified behavior (Klein et al., 2014), but when they do not find sufficient explanation, they nonetheless infer that the person likely had *some* reason justifying what they did—and imagine possibilities of what it could have been. We believe that the ability to infer phantom costs is a facet of social cognition that can serve as a protective mechanism, preventing individuals from falling victim to scams by prompting them to contemplate reasons for unusually generous offers, one of which might be a phantom cost associated with a scam. Future research should explore how phantom costs influence various economic aspects, including their role in susceptibility to scams, fraud, and online deception (catfishing).

### Constraints on Generalizability

Our model predicts phantom costs occur under specific conditions: People are expected to follow the norm of self-interest, someone violates that norm of self-interest, and there is no other reason justifying and explaining the norm violation. The more a situation deviates from these conditions, the less likely someone is to imagine phantom costs. Thus, we would expect phantom costs in exchange relationships, where self-interest is the norm, but not in communal relationships, because acts of generosity and selflessness are customary and justified in such settings (Clark & Mills, 1993). Similarly, when sufficient explanation for behavior is readily available, people should not look for phantom costs to explain it. Thus, phantom costs occur in specific conditions of unjustified or unexplained generosity.

But what counts as unjustified or unexplained generosity? Such judgments are subjective and personal, depending on an individual’s values and beliefs about what kinds of things are sufficient to motivate another person. Thus, different people should respond differently to offers—even identical offers—if they think differently about what a sufficient explanation would be. Future research should examine how individual differences may moderate phantom costs and monetary backfires.

Phantom costs occur because of mind perception: People imagine phantom costs to try to understand another person’s mind. Thus, we would expect people with psychopathological deficits in mind perception of agency including autism or schizotypy (Gray et al., 2011) would be less likely to imagine phantom costs. Similarly, young children who have not yet developed complex mind perception abilities (Flavell, 1999) might not imagine phantom costs. Some situational factors like cognitive load distractors or stressors undermining mind perception might also reduce phantom costs (Waytz et al., 2010). Thus, deficits in mind perception abilities should reduce phantom costs.

Relatedly, offers from agents perceived to lack important aspects of mind should not generate phantom costs. Offers from nonhuman agents might generate fewer phantom costs, to the extent they are seen as non-agentic—for example, if a robot or artificial intelligence is overly generous, people may not imagine phantom costs if they do not believe it is a conscious, self-interested agent (Mandell et al., 2017). Similarly, offers from young children who do not understand the value of money, or from foreigners who do not understand cultural conventions around bargaining or the value of the local currency might not generate phantom costs because these are sufficient reasons for their overly generous offers.

Our model specifies when phantom costs produce money backfires: When it reduces the net psychological value of an exchange. Thus, money backfires should not occur when people fail to imagine phantom costs or they think the extra money is “worth the risk.”

Across various types of wage-labor scenarios, our research revealed a consistent pattern: moderately generous wages increased job interest, but excessively high wages led to a decrease in interest. Further investigation is necessary to determine whether this same pattern holds in different contexts, such as varying product discounts (e.g., 30%, 50%, 90%, 99%). It’s important to note that our model doesn’t provide precise predictions for how individuals will respond to specific offers because the net psychological value hinges on subjective judgments that are challenging to predict beforehand. Nevertheless, our model strongly asserts that the success or failure of monetary incentives depends on this net psychological value, which involves elements that are influenced by the unique characteristics of an individual’s mind and values. For example, a person who imagines phantom costs might still accept a transaction because they are unusually risk-seeking, don’t care about money, are feeling especially generous, or other idiosyncratic values. Nonetheless, features like this would be part of their computation of net psychological value.

The present studies investigated this phenomenon in the United States and Iran. It is conceivable there could be cultural differences in this phenomenon, but if there are we expect they should fit within our model. For example, the few isolated cultures in which people have not been exposed to market norms and the norm of self-interest might not show this effect—which our model would explain via a lack of the norm of self-interest. Similarly, in some cultures people regularly engage in overly generous behaviors, such as the *potlatch* tradition among Northwest Native Americans in which people could gain status by giving away or destroying valuable objects. In these cultures, people might not imagine phantom costs, but our model would explain this because the potlatch tradition provides justifying reasons for this overly generous behavior. We suspect, however, that even in cultures where giving away money is a main means of gaining status, there is likely some version of phantom costs: For example, people might find it suspicious if someone were willing to overly generously lose status by *accepting* an overly generous economic offer.

## Conclusion

Refusing a job offer that pays too much, rejecting an inexpensive plane ticket, and not eating a stranger’s cookie are all different effects. However, each represents cases in which offers are rejected, not because people offered too little, but because they offered too much. These studies provide a simple but comprehensive reason for when and why monetary incentives can backfire—because people infer phantom costs that outweigh their excitement for more money. Money backfires are surprising from the perspective of the standard economic model because it assumes people make decisions based on the value of exchanges: People usually take the option with higher (monetary) value. Instead, however, people make these decisions based on the *psychological value* of the options, and phantom costs can mean that the monetary value and the psychological value move in opposite directions—offering more money can reduce the psychological value of an offer. Phantom costs thus both help redeem and call into question the standard model, suggesting that people are more rational than many believe, but also that market exchanges are more social than often thought. Behind economic exchanges lurk the minds of people who want money but who are also think about the motivations of their exchange partners. The key is that money is not only bits of paper or numbers in a bank account but also a signal of what someone wants—and what they may be hiding.
